# Interaction of PKR with single-stranded RNA

**DOI:** 10.1038/s41598-017-03047-7

**Published:** 2017-06-13

**Authors:** Christopher B. Mayo, James L. Cole

**Affiliations:** 1 Department of Molecular and Cell Biology, University of Connecticut, Storrs, Connecticut 06269 USA; 2 Department of Chemistry, University of Connecticut, Storrs, Connecticut 06269 USA

## Abstract

Although the antiviral kinase PKR was originally characterized as a double-stranded RNA activated enzyme it can be stimulated by RNAs containing limited secondary structure. Single-stranded regions in such RNAs contribute to binding and activation but the mechanism is not understood. Here, we demonstrate that single-stranded RNAs bind to PKR with micromolar dissociation constants and can induce activation. Addition of a 5′-triphosphate slightly enhances binding affinity. Single-stranded RNAs also activate PKR constructs lacking the double-stranded RNA binding domain and bind to a basic region adjacent to the N-terminus of the kinase. However, the isolated kinase is not activated by and does not bind single-stranded RNA. Photocrosslinking measurements demonstrate that that the basic region interacts with RNA in the context of full length PKR. We propose that bivalent interactions with the double stranded RNA binding domain and the basic region underlie the ability of RNAs containing limited structure to activate PKR by enhancing binding affinity and thereby increasing the population of productive complexes containing two PKRs bound to a single RNA.

## Introduction

Protein Kinase R (PKR) is a serine/threonine kinase which plays a critical role in the innate immunity pathway. PKR is synthesized in a latent form and is activated by duplex regions present within structured RNAs to undergo autophosphorylation. Stimulatory RNAs typically originate from viral infection but several endogenous RNAs have been identified as PKR activators^1–5^. PKR phosphorylates its primary cellular substrate, the α-subunit of eukaryotic initiation factor-2 (eIF2α), to inhibit protein synthesis and viral replication in infected cells^6^.

**Figure 1:**
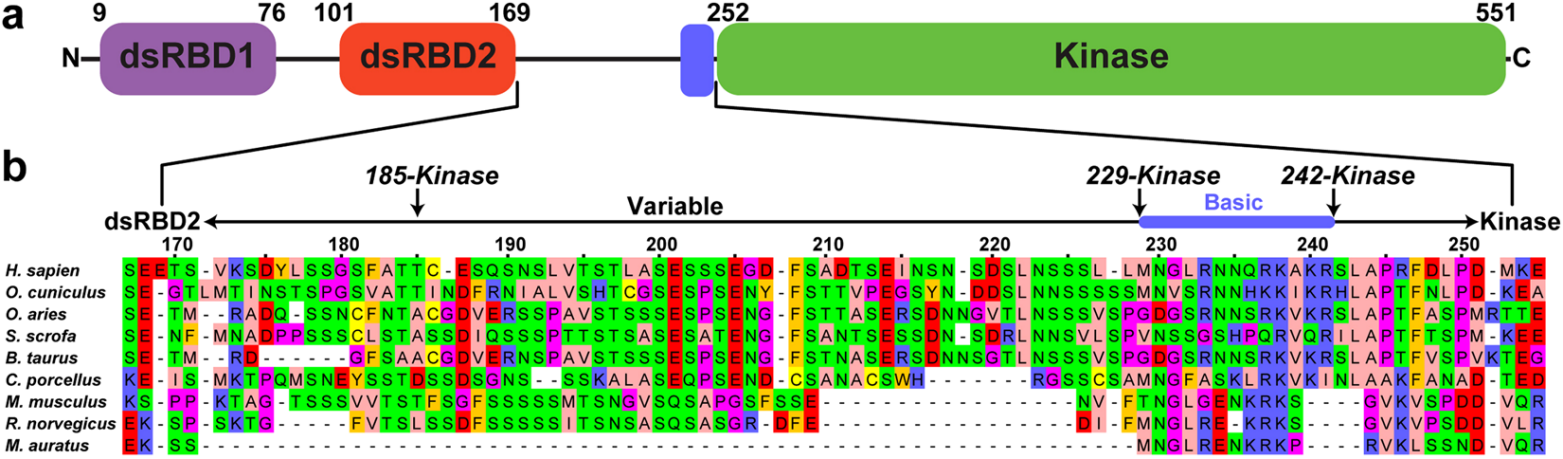
PKR domain schematic. (**a**) PKR domain organization. Canonical domain boundary positions are indicated above each domain. (**b**) Linker sequence alignment. The alignment was generated with ClustalW^55^ and prepared in JalView^56^. Residues are colored using the Zappo coloring scheme.

PKR is comprised of two tandem N-terminal dsRNA binding domains (dsRBD1 and dsRBD2) and a C-terminal kinase domain connected by an ~80 residue flexible linker (Fig. 1). The dsRBD recognizes an A-form RNA duplex with a site size of ~15 bp and primarily makes interactions with 2′-hydroxyls and the phosphodiester backbone^7, 8^. Both dsRBDs in PKR adopt a canonical αβββα fold^9^. The kinase domain has a bilobal architecture typical of protein kinases consisting of a smaller N-terminal lobe and larger C-terminal lobe connected by a flexible hinge region. The kinase domain co-crystallizes with eIF2α as a back to back dimer with an allosteric pathway linking the dimer interface to the active site^10^. Biochemical and biophysical data indicate that dimerization plays a critical role in the PKR activation mechanism^11^. Mutations to key residues within the dimer interface abolish activation^12^. Activation of PKR by simple dsRNAs requires a minimum of 30 bp^13, 14^ and is mediated by dimerization of the kinase domain^15^.

In addition to the canonical activators containing ≥30 bp dsRNA regions, PKR is stimulated by RNAs that contain limited secondary structure and include single-stranded regions or tertiary structure. Examples include a 17 bp stem loop within the 3′-UTR of TNF-α mRNA^16^, a pseudoknot within the 5′-UTR of IFN-γ mRNA^4, 5^, siRNAs containing short ssRNA overhangs^17, 18^, several snoRNAs induced under metabolic stress^3^, and certain bacterial RNAs^19^. Notably, short stem-loop RNAs with flanking ssRNA tails (ss-dsRNAs) have been identified as a potent PKR activation motif^20, 21^. Truncations to the ssRNA regions result in decreased binding affinity and activation potency, and complete deletion of either the 5′- or 3′- flanking tail abolishes activation^22^. Surprisingly, a duplex region as short as 10 nt with 15 nt 5′- and 3′- flanking tails binds two PKR monomers and functions as an activator. These observations imply that single-stranded regions can play a role in RNA-mediated activation of PKR. Although PKR does not contain a canonical ssRNA binding domain, interactions between ssRNAs and dsRBDs from other proteins has been reported^23, 24^. However, mutations in PKR within the dsRBDs that block dsRNA binding do not prevent photocrosslinking to an RNA with minimal secondary structure, suggesting that ssRNA binding may be mediated by other regions of the enzyme^25^. Potential interaction sites include a basic region adjacent to the N-terminus of the kinase (Fig. 1) that is important for kinase function^26^ as well as a basic cleft lying on the kinase domain^27^. A 5′-triphosphate (5′-ppp) is reported to be critical for PKR activation by RNAs with limited secondary structure, including the ss-dsRNA motif^3, 19, 21, 28^ (but see ref. 22). Duplex RNAs do not exhibit a triphosphate dependence so it is likely that ssRNA and the 5′-ppp bind to the same site^21^.

Here, we have directly probed ssRNA binding and activation of PKR. We demonstrate that ssRNAs can bind to PKR at both the dsRBD and the basic region. ssRNA mediated activation of the kinase domain requires the basic region. Our data support a model where PKR activation by RNAs is regulated *in vivo* by interaction with both duplex and single-stranded regions.

## Results and Discussion

### PKR interaction with ssRNA

**Figure 2:**
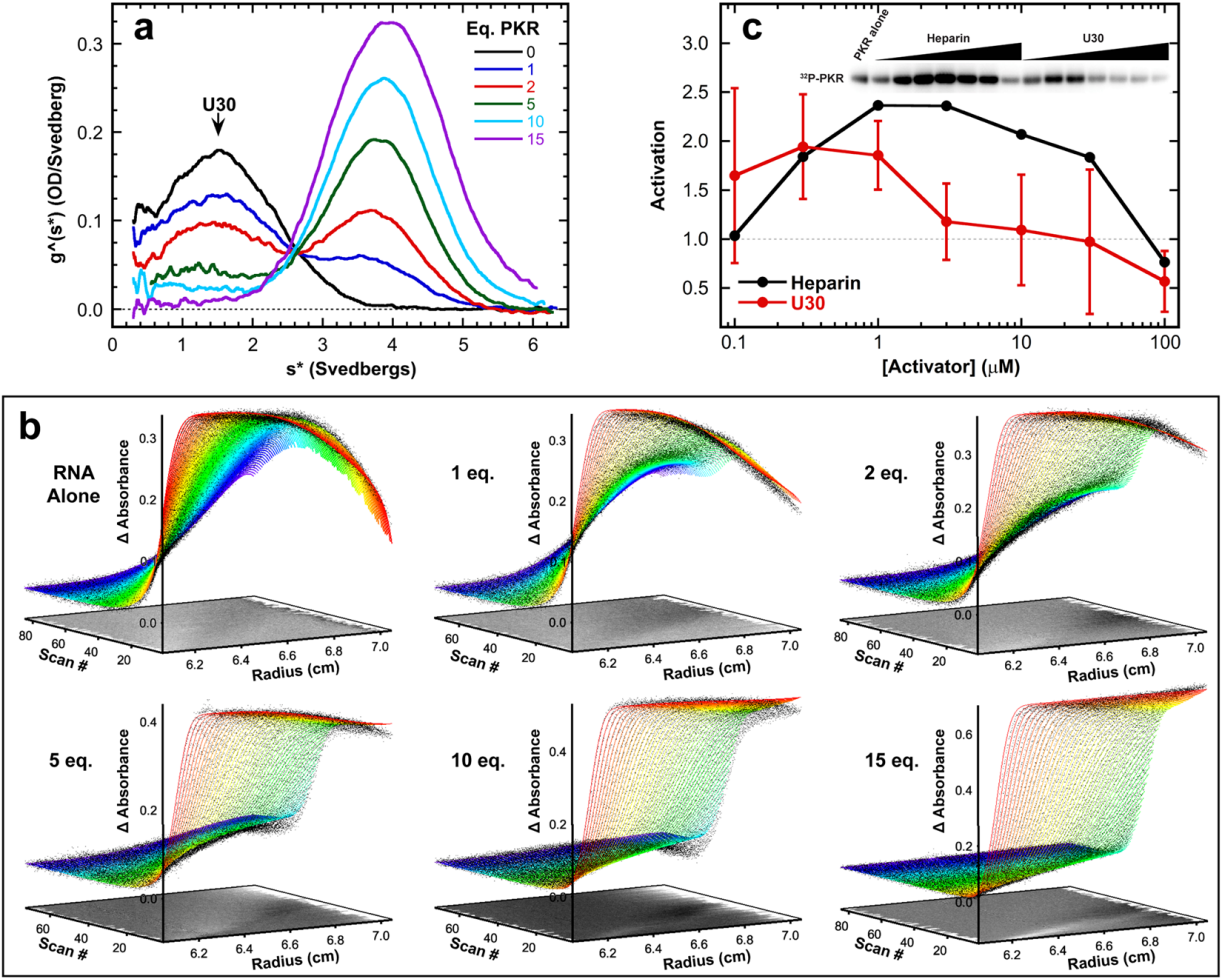
Interaction of PKR with ssRNA. PKR binding to U30 ssRNA was assayed by sedimentation velocity analytical ultracentrifugation. Measurements were performed in AU75 buffer at 20 °C and 50,000 rpm using absorbance detection at 260 nm. (**a**) Titration of U30 with PKR represented as an overlay of g^(s*) sedimentation coefficient distribution functions. The samples contained 1 µM U30 () and 1 µM U30 plus 1 eq. (), 2 eq. (), 5 eq. (), 10 eq. (), and 15 eq. PKR (). The decrease in the U30 peak and appearance of the peak at higher S are due to complex formation. (**b**) Global analysis of the time difference curves. Scans within each data set were subtracted in pairs to remove time-invariant background noise and fit to a sequential 2:1 binding model using SEDANAL^53^. The data are indicated by points and the fit by solid lines. The residuals are plotted as a grayscale image in the x-y plane at z = 0. The best-fit parameters are in Table 1. (**c**) Activation of PKR by U30. 500 nM PKR was incubated with variable concentrations of U30 in AU75 buffer with 5 mM MgCl_2_ for 20 min at 32 °C. Samples were resolved by SDS-PAGE and ^32^P-PKR was quantified with a phosphorimager. The data are normalized to activation of PKR in the absence of activator. The error bars correspond to the standard deviation from three replicates. The inset shows a cropped image of the gel.

Based on the critical contribution of single-stranded regions to the binding and activation of PKR by ss-dsRNAs, we have investigated the interactions of PKR with isolated ssRNAs. We employed sedimentation velocity analytical ultracentrifugation in order to detect transient, lower affinity interactions that may not be reliably measured in gel shift^29^ or filter binding^30^ assays. Figure 2a shows a titration of a model ssRNA, U30, with PKR depicted as an overlay of g^(s*) sedimentation coefficient distribution functions. The homopolymeric U30 RNA was chosen to avoid base stacking and potential formation of secondary structure. Addition of PKR results in a decrease in the amplitude of the peak at 1.5S associated with free U30 and formation of a peak at higher sedimentation coefficient due to complex formation.

In order to define the interaction stoichiometry and obtain dissociation constants the sedimentation velocity profiles were subtracted in pairs to remove systematic noise and the resulting difference curves were fit to alternative association models. The data are well described by a 2:1 sequential binding model where two PKR monomers assembly on a single RNA (Fig. 2b). Similar to PKR interactions with duplex RNAs, the first PKR binds with the highest affinity, with a *K*_d1_ = 3.5 µM, and the second binds weaker, *K*_d2_ = 40 µM (Table 1). Nonspecific, sequential binding of multiple proteins to a nucleic acid lattice of identical sites, such as U30, is expected to become progressively weaker due to statistical effects^31^. Binding to U30 activates PKR autophosphorylation weakly (Fig. 2c) to a level about 1.5-fold above the background in the absence of RNA. As observed with dsRNA, higher ssRNA concentrations inhibit, consistent with dilution of PKR dimers by the excess nucleic acid. PKR binds about three-fold more strongly to a heteropolymeric ssRNA of the same length (Het30, Table 1). This enhancement may reflect weak secondary structure formation by the heteropolymeric sequence (Fig. S1) or some slight nucleotide preference.

**Table 1:** Sedimentation velocity analysis of PKR constructs binding to RNA.

Protein	Nucleic Acid	*K*_d1_ (µM)	*K*_d2_ (µM)	RMSD^a^
Full length	U30	3.51 (3.49, 3.52)	39.9 (39.4, 40.5)	0.00662
	ppp-Het30	0.825 (0.761, 0.894)	3.81 (3.49, 4.15)	0.00876
	Het30	1.07 (1.01, 1.12)	10.8 (10.1, 11.6)	0.00667
dsRBD	U30	8.42 (7.98, 8.88)	52.3 (47.6, 57.7)	0.00514
	ppp-Het30^b^	5.36 (4.60, 6.31)	9.66 (8.03, 11.6)	0.00810
	Het30^b^	8.21 (7.38, 9.18)	19.4 (16.3, 23.0)	0.00701
185-kinase	U30	31.9 (29.8, 34.2)	68.9 (51.8, 97.5)	0.00452
229-kinase	U30	1.91 (1.79, 2.05)	7.87 (7.16, 8.64)	0.00706
	ppp-Het30	ND^c^	ND^c^	ND^c^
	Het30	ND^c^	ND^c^	ND^c^
	ds30	3.80 (3.37, 4.33)	3.27 (2.82, 3.77)	0.00457
	dT30	2.59 (2.44, 2.76)	16.2 (14.5, 18.2)	0.00630
242-kinase^d^	U30	90.0 (84.2, 96.5)	—	0.00546
	ds30	19.9 (18.6, 21.4)	—	0.00457

Parameters obtained by global nonlinear least square analysis of the sedimentation velocity data using a model of sequential binding of two proteins monomers. The values in parentheses represent the 95% joint confidence intervals obtained using the F-statistic.

^a^Root mean square deviation in absorbance units.

^b^Good fits required that the sedimentation coefficients for the protein-RNA complexes be allowed to float to their best-fit values.

^c^Not determined. More than two protein monomers bind to the RNA and the data could not be reliably fit to an association model. Plots of the normalized g^(s*) distributions for these experiments are in Fig. S3.

^d^242-kinase data were fit to a 1:1 binding model.

These data provide the first direct evidence that PKR binds to and is activated by single-stranded RNAs and rationalizes previous reports^19, 21, 22^ that single-stranded regions present in structured RNAs contribute to PKR interactions. As in the case of duplex RNAs^32^, the bell-shape activation curve implies that ssRNAs induce PKR dimerization. The low extent of activation is likely a consequence of the relatively weak binding affinity and it is unlikely that PKR is significantly activated *in vivo* upon interaction with unstructured RNA.

### Localization of ssRNA binding

We characterized U30 and Het30 binding to individual PKR domain constructs to define the region(s) responsible for interaction with ssRNA and the contribution of the 5′-ppp. The dsRBD (residues 1–184) binds U30 with affinities slightly reduced relative to the holoenzyme (Table 1), indicating that ssRNA can also bind to this canonical dsRNA binding site. However, there are other potential ssRNA binding sites in PKR. In the human enzyme, the dsRBD is separated from the kinase domain by an ~80 residue unstructured linker. The length of the linker is highly variable among PKR orthologs (Fig. 1b). This length variability arises within the N-terminal acidic portion of the linker, but a cluster of basic residues within the C-terminal portion is conserved. This portion of the linker is implicated in kinase function^26^ and ribosome association^33^ and represents a potential RNA interaction site. Alternatively, the heparin binding site is located on the kinase domain^27, 34, 35^ and represents another possible ssRNA binding motif. We prepared PKR domain constructs to probe the role of the linker, basic region, and isolated kinase domain in ssRNA binding. Each of the constructs is homogeneous and monomeric up to the highest concentration assayed (Fig. S2, Table S1).

**Figure 3:**
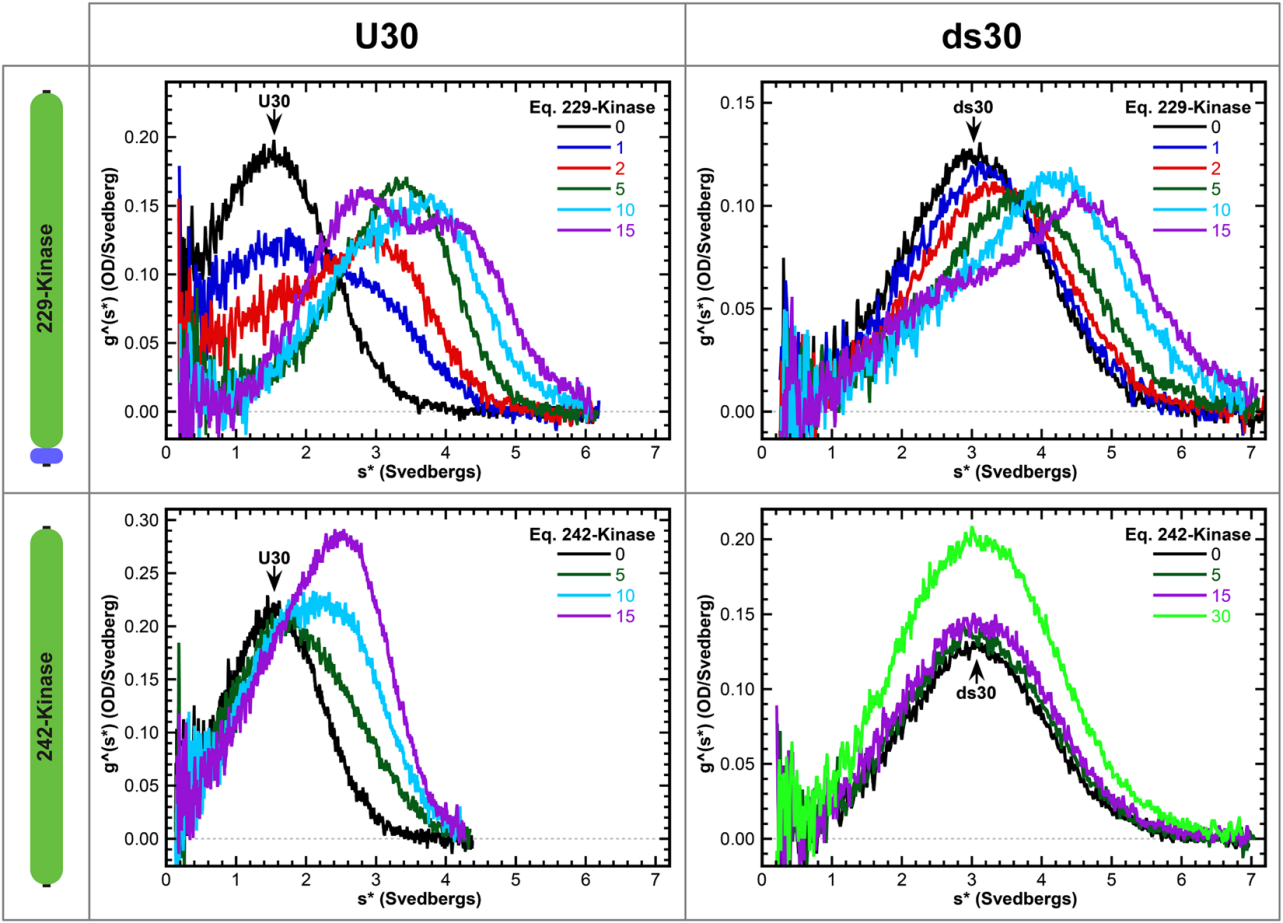
Interaction of the PKR kinase domain/basic region with RNA. Binding of 229-kinase and 242-kinase to U30 and ds30 were analyzed by sedimentation velocity. Measurements were performed in AU75 buffer at 20 °C and 50,000 rpm using absorbance detection at 260 nm. Each panel shows a titration of RNA with protein depicted as a g^(s*) sedimentation coefficient distribution. Addition of 229-kinase to U30 or ds30 causes a shift in the distributions to the right, indicating complex formation. In contrast, 242-kinase induces only a slight decrease in the peak corresponding to free U30, indicating a very weak interaction. The peak at 2.8 S at higher protein concentrations is due to free 242-kinase. The sedimentation coefficients of 242-kinase and ds30 are similar and the increase in the peak amplitude is primarily due to free protein. 229-kinase data were fit to a sequential 2:1 binding model and 242-kinase data were fit to a 1:1 binding model to extract the binding parameters displayed in Table 1.

Figure 3 shows titrations of U30 and ds30 RNAs with PKR kinase constructs that contain (229-kinase) or lack (242-kinase) the basic region. Addition of stoichiometric 229-kinase to U30 causes a decrease in the RNA peak and a shift of the distribution to higher sedimentation coefficient, indicating that this construct binds to ssRNA. The distribution at the highest protein concentration is bilobal due to the contribution of free 229-kinase at 2.8S and a protein:RNA complex which sediments at 4.7S. In contrast, addition of the 242-kinase construct lacking the basic region results in only a minimal decrease in the free RNA peak. The new feature which appears at 2.8 S is primarily due to absorbance of the free protein. The 229-kinase data fit well to the sequential 2:1 binding model with *K*_d1_ = 1.9 µM and *K*_d2_ = 7.9 µM. However, the 242-kinase binds very weakly and binding of a second protein was not detected. The data were fit to a 1:1 model (Table 1). Thus, the basic region mediates ssRNA binding to the C-terminal portion of PKR. Affinity is substantially reduced when the remainder of the linker is included in the 185-kinase construct. These measurements may be more representative of the affinity of the basic region for ssRNAs in the context of full length PKR.

Interestingly, 229-kinase also binds a 30 bp dsRNA and the dissociation constants are similar to those observed with U30. Thus, the basic region interaction is not specific for ssRNA. Indeed, 229-kinase also binds to a 30 nt ssDNA, dT30, albeit with reduced affinity indicating a small contribution from the 2′-OH (Table 1). The 242-kinase and ds30 have similar sedimentation coefficients (3.2 and 2.8S, respectively) so that the two species are not resolved by time derivative analysis. Although a discrete peak for the complex is not detected, the data are best described by a 1:1 binding model. The affinity is weaker than for 229-kinase.

Given previous reports indicating that a 5′-ppp contributes to PKR activation to certain RNAs with limited secondary structure^3, 19, 21, 28^, we probed the effect of this moiety on PKR binding to an ssRNA, Het30. The presence of a 5′-ppp enhances PKR binding, reducing *K*_d2_ by a factor of about 3 relative to the 5′-OH RNA (Table 1), but the binding affinities are still weaker than for a 30 bp dsRNA of the same length^36^. A similar magnitude effect of a 5′-ppp is observed for Het30 binding to the dsRBD. It was not possible to fit the data for binding of 229-kinase to Het30 and ppp-Het30 due to the formation of higher-order complexes (Fig. S3). However, comparison of the g^(s*) distributions at comparable protein concentrations with Het30 and ppp-Het30 reveals that the 5′-ppp slightly increases binding to both PKR domain constructs. Thus, the enhancement of ssRNA binding to PKR by the 5′-ppp is not associated with a distinct binding site, as previous suggested^25^ but is nonspecific and likely attributable to electrostatic interactions. A 5′-ppp also increases the affinity in the context of a ss-dsRNA containing a 15 bp stem and 15 nt 5′- and 3′-tails (15-15-15) but it is not required to detect kinase activation^22^. A 5′-ppp may play a role in PKR activation by RNAs with limited secondary structure^3, 19, 21, 28^ by increasing binding affinity and thereby increasing the maximum population of active PKR dimers. However, a 5′-ppp is not absolutely required for activation of PKR, or the 229-kinase (*vide infra*), by unstructured RNAs.

The weak dependence of binding affinity on a 5′-ppp is consistent with the absence of a distinct binding site for this moiety in PKR. The canonical cytosolic sensor for RNAs bearing a 5′-ppp is RIG-I. This protein contains a helicase domain which binds duplex RNA and a regulatory domain which binds 5′-ppp^37^. For RIG-I, the presence of a 5′-ppp increases the binding affinity by >100-fold relative to the 5′-OH form, but the enhancement is only 2.4-fold for the isolated helicase domain^38^. The latter is similar in magnitude to PKR and presumably represents the contribution of nonspecific electrostatic interactions. The antiviral proteins IFIT1 and IFIT5 recognize ssRNAs containing a 5′-ppp via a deep, positively-charged cavity^39, 40^. IFIT5 binds a 5′-ppp ssRNA with nanomolar affinity yet does not form a detectable complex with ssRNA bearing a 5′-OH^41, 42^.

**Figure 4:**
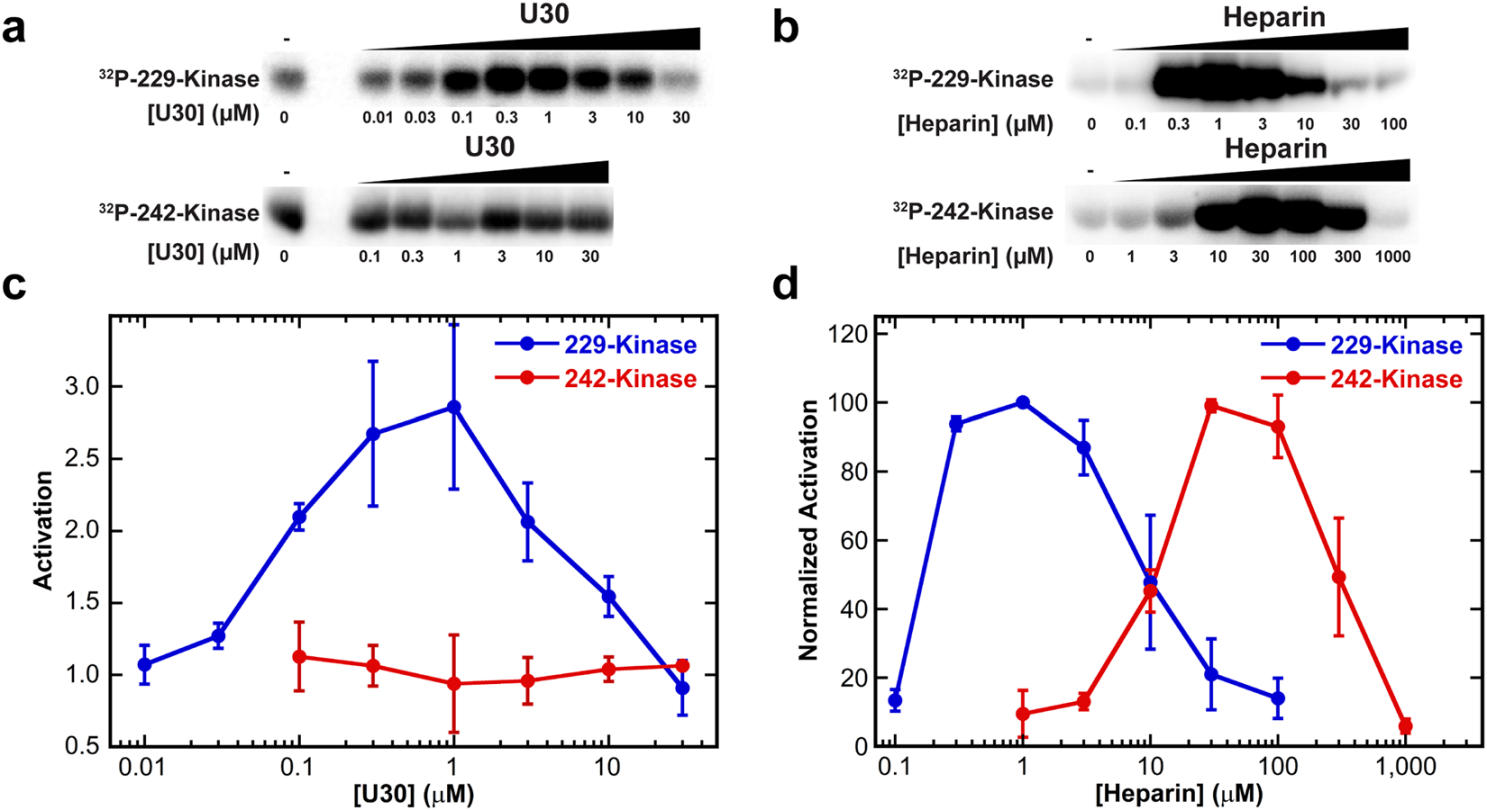
Activation of PKR kinase domain by ssRNA. Autophosphorylation reactions were carried out with a fixed concentration of protein and titrated with either U30 (**a**,**c**) or heparin (**b**,**d**). The basal (RNA-independent) activities of 229- and 242-kinase are much lower than the full length enzyme and are different from each other (Fig. S4). Thus, the protein concentrations were adjusted to give approximately equal extents of autophosphorylation in the absence of RNA: 1.5 µM 229-kinase and 5 µM 242-kinase. Phosphorimager scans of SDS-PAGE gels are shown in (**a**) and (**b**). The gels images are cropped to display the ^32^P kinase domain bands and the contrast settings within each gel are independently adjusted using using ImageQuant TL software to clearly show the intensity trends. The quantitation of ^32^P-incorporation is shown in (**c**) and (**d**). The error bars correspond to the standard deviation from three replicates. In (**c**), the activation by U30 is plotted relative to samples containing no activator. In (**d**), activation by heparin is normalized to the maximum signal because heparin is a potent activator and quantitation relative to the low signal in the absence of activator is not accurate. The ~100-fold increase in the heparin concentration required for maximal activation of the 242-kinase persists when the protein concentration is reduced to the same concentration used for 229-kinase (1.5 µM). Note that the maximum concentration of U30 assayed was 30 µM which is the peak of the 242-kinase heparin activation curve.

Given the strong binding of ssRNA to 229-kinase we asked whether it could mediate activation. For comparison, we also assayed activation of the 242-kinase construct. The 229-kinase is activated by U30 with a bell-shaped activation curve (Fig. 4a and c). As observed for the full length enzyme, the maximum extent of activation by ssRNA is fairly weak, corresponding to about 3-fold over the basal level. Consistent with the lower ssRNA binding affinity, 242-kinase is not activated by U30.

In a previous study we localized the heparin binding site to a pocket adjacent to helix αC within the kinase domain using an inactive construct corresponding to 242-kinase^27^. Figure 4b and d show that both 229- and 242-kinase are activated by heparin. The peak of maximal activation is shifted to about 100-fold lower heparin concentration for the 229-kinase implying that the basic region may also interact with heparin. Consistent with this interpretation, heparin binds more weakly to 242-kinase than to the full length enzyme^27^. The presence of the basic region contributes to heparin-mediated activation and possibly binding affinity. However, the heparin binding pocket on the kinase domain does not accommodate single-stranded RNA.

**Figure 5:**
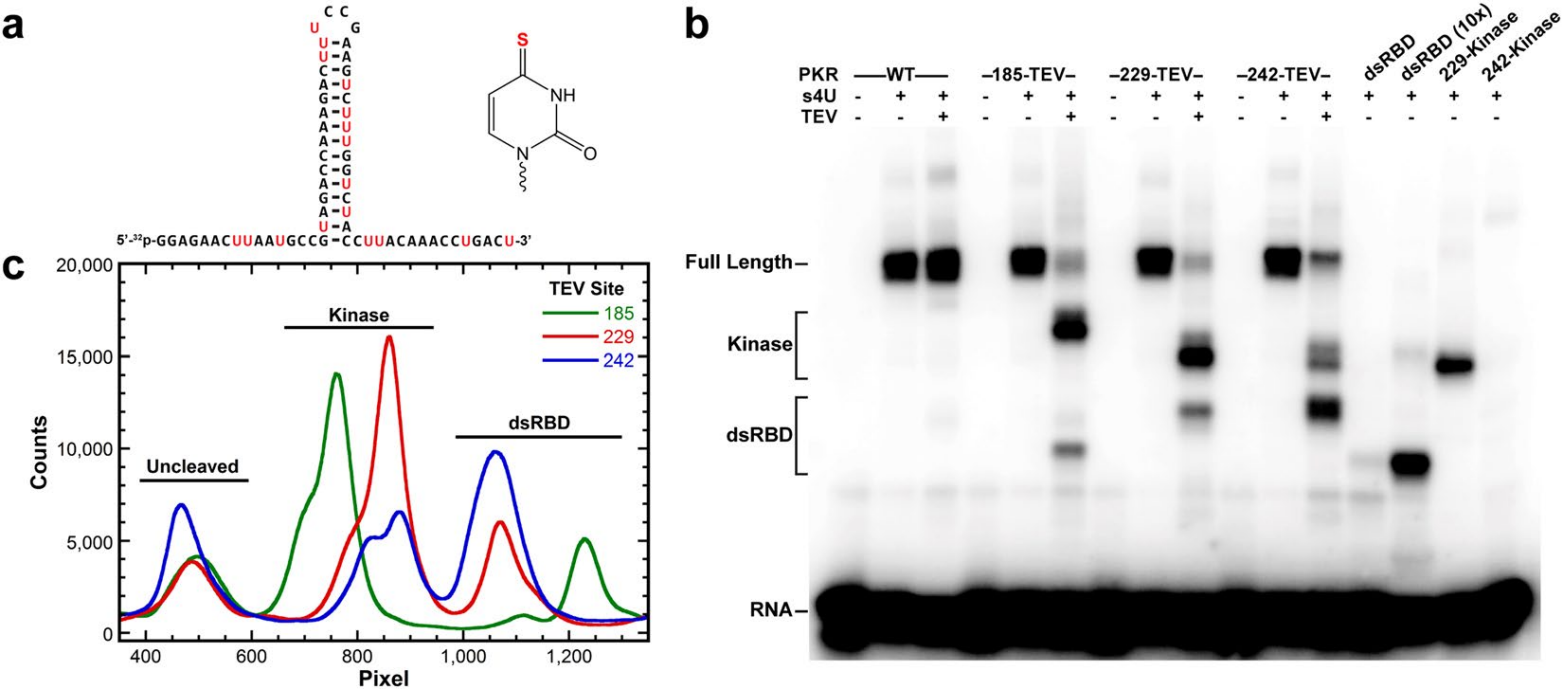
Crosslinking analysis of PKR binding to ss-dsRNA. (**a**) Secondary structure of the ss-dsRNA (15-15-15) and chemical structure of 4-thiouridine. Modified uridines are highlighted in red and the sulfur responsible for photochemical crosslinking is highlighted in red in the 4-thiouridine structure. (**b**) Phosphorimager analysis of ^32^P incorporation in RNA:protein complexes following crosslinking. Full length PKR constructs containing a TEV protease site and individual domains were crosslinked to radiolabeled ss-dsRNA. TEV cleavage was performed after the crosslinking reaction. The P1′ position of the cleavage site is indicated above the lanes. Samples contained 0.5 µM RNA and 1 µM protein. One sample of dsRBD contained 10 µM protein (labeled 10x). Lanes labeled as (−) s4U contained unmodified ss-dsRNA. (**c**) Quantitation of RNA:protein adducts following TEV cleavage. Distributions were created by vertical integration of data from panel b.

Our results indicate that PKR contains two RNA binding loci. We performed photocrosslinking assays to provide insight into how these regions interact with RNA in the context of the full length enzyme and 15-15-15, a potent ss-dsRNA activator. The ss-dsRNA was transcribed in the presence of 4-thiouridine triphosphate (s4U), resulting in incorporation of the modified nucleotide throughout the RNA (Fig. 5a), radiolabeled at the 5′-end and crosslinked to PKR by exposure to 365 nm light. Reactions were performed with PKR domain constructs and full length PKR constructs containing a TEV protease cleavage site located at position 185, 229, or 242. Following crosslinking, samples were cleaved by TEV protease, separated on SDS-PAGE and visualized by phosphorimaging. Crosslinking of the ss-dsRNA to wild type PKR is dependent on s4U and the adduct is not cleaved by TEV protease (Fig. 5b). The mutants containing TEV sites are efficiently cleaved by the protease giving two predominant products. When cleaved at positions 185 or 229 that are N-terminal to the basic region, most of the ^32^P-labeled RNA is associated with the C-terminal kinase domain. Cleavage at position 242 shifts the distribution so most of the label is attached to the N-terminal fragment containing the dsRBD and basic region. This switch in band intensity indicates that the basic region interacts with the ss-dsRNA in the context of full length PKR. The residual crosslinking of the C-terminal fragment may reflect the weak interactions observed between the 242-kinase construct and RNA.

Crosslinking of the ss-dsRNA to the isolated domain constructs mirrors the trends observed with the full length protein. The kinase domain requires the basic region for crosslinking. Crosslinking of ss-dsRNA to the dsRBD is weak and requires addition of 10-fold molar excess of protein relative to the other samples to generate a similar amount of adduct. This low amount of product is not due to weak binding but likely reflect limited accessibility of s4U when the dsRBD is bound to dsRNA, its preferred binding site. The photoreactive thiol group lies in the major groove and dsRBDs do not interact with the major groove face of bases in the dsRNA helix^7^. The dsRBD binds duplex RNA strongly (intrinsic *K*_d_ ~ 100 nM)^31^ whereas the affinity toward single-stranded RNA is in the low micromolar range (Table 1). Thus, the isolated dsRBD populates the single-stranded regions only after the duplex has been saturated, giving rise to more efficient crosslinking to s4U. In the context of the full length protein complex formation is driven by the high affinity interaction of the dsRBD with the duplex region. The crosslinking data indicate that the auxiliary interactions which stabilize the complex occur between the single-stranded tails and the basic region.

We have tested this model by extending our previous study of ss-dsRNAs^22^ to analyze the binding contribution or single-stranded regions in the context of the isolated dsRBD. Binding affinities were obtained for 15-15-15 and a construct lacking the flanking tails, 0-15-0. The measurements are reported in Table 2 along with the previously-measured^22^ affinities for the full length protein. The relative binding affinities indicate that the tails predominantly interact with regions outside of the dsRBD. Removal of the tails decreases the affinity of full length PKR by about 35-fold but reduces dsRBD binding by only about two-fold. In the absence of the tails the two constructs bind about equally well, confirming that binding to duplex regions is mediated primarily by the dsRBD.

**Table 2:** Sedimentation velocity analysis of PKR and dsRBD binding to ss-dsRNAs.

Protein	RNA^a^	*K*_d1_ (nM)	*K*_d2_ (nM)	*K*_d3_ (nM)	RMSD^b^
PKR	15-15-15	4 (2, 6)	294 (252, 343)	—	0.00821^c^
PKR	0-15-0	138 (105, 178)	1660 (1310, 2130)	—	0.00906^c^
PKR	15-5-15	118 (93, 150)	853 (673, 1103)	—	0.00667^c^
PKR	0-5-0	5400 (4930, 5900)	—	—	0.00460
dsRBD	15-15-15	111 (76, 160)	190 (121, 279)	2780 (2100, 3930)	0.00358
dsRBD	0-15-0	218 (155, 300)	1097 (961, 1249)	—	0.00493

Parameters obtained by global nonlinear least square analysis of the sedimentation velocity data. The values in parentheses represent the 95% joint confidence intervals obtained using the F-statistic.

^a^The three numbers indicate the length of the 5′-tail (nt), the stem (bp), and the 3′-tail (nt), respectively. Each of the RNAs contains a 5′-ppp and a tetraloop capping the stem sequence.

^b^Root mean square deviation in absorbance.

^c^Data from ref. 22.

The relatively weak binding of ssRNAs to PKR and the modest activation indicates that isolated single-stranded RNAs are unlikely to form a stable complex with PKR or to promote significant activation *in vivo*. However, these interactions provide a rationale for the contribution of single-stranded regions to PKR binding and activation in the context of structured RNAs. Invariably, potent RNA activators of PKR possess some secondary and tertiary structure^43^. Some of these RNAs lack the minimum 30 bp of duplex required to support PKR activation by simple dsRNAs but can form extended double stranded regions by coaxial stacking of shorter helices^5^ or by dimerization of self-complementary stem loops^44, 45^. Other RNA activators contain a single duplex as short as 10–15 bp^21, 22^ or two hairpins of 5 and 4 bp^21^, implying a substantial interaction of PKR with the unstructured regions. The crosslinking results demonstrate that both the dsRBD and the basic region-kinase domain interact with RNAs containing structured and unstructured regions. Despite the presence of these two RNA binding sites, sedimentation velocity data for full length PKR binding to ssRNAs and ss-dsRNAs only fit to a model where two monomers sequentially assemble onto a single RNA. The data do not fit well to alternative models which incorporate binding of multiple RNA ligands to a single protein (see supplement). The ~80 residue region separating the dsRBD and the basic region (Fig. 1) is intrinsically disordered and PKR adopts multiple conformations in solution^46, 47^. NMR data indicate that dsRBD2 can transiently interact with the kinase domain^48, 49^ suggesting a model in which PKR exists in an equilibrium between open and closed states^50^. In the closed conformation, the dsRBD and basic region/kinase domain would be in close proximity and capable of binding to a single RNA.

We propose that bivalent interactions underlie the ability of RNAs containing limited structure to activate PKR by enhancing binding affinity and thereby increasing the population of productive complexes containing two PKRs bound to a single RNA. In this model, a major contribution driving complex formation is the well-characterized interaction of duplex regions with the dsRBD^31, 36^. This interaction is stabilized by adjoining unstructured regions binding to the basic region. The bivalent interaction gives rise to strong (*K*_d_ ~ 100 nM) binding even for ss-dsRNAs containing a stem as short as 5 bp. In addition to enhancing binding, the bivalent interactions may serve to orient the kinase domains to increase the propensity for the formation of active dimers^15^.

## Methods

### Protein expression and purification

PKR kinase domain constructs were created by inserting a TEV protease cleavage site (ENLYFQ↓G/S) upstream of the desired N-terminus. The 185- and 229-kinase domain constructs contain an extra glycine at the N-terminus. Wild-type PKR and constructs containing a TEV protease cleavage site were expressed and purified as previously described^27^. Following elution from the heparin sepharose column, the constructs containing a cleavage site were treated with AcTEV protease (Thermo Fisher Scientific) overnight at 4 °C. The kinase domain constructs were purified on hydroxyapatite (CHT ceramic hydroxyapatite, Bio-Rad Laboratories, Inc.). The dsRBD (residues 1–184) was expressed and purified as previously described^31^. All proteins were further purified by size exclusion chromatography on Superdex 75 or 200 HiLoad 16/60 columns (GE Healthcare) into AU75 buffer (75 mM HEPES (pH 7.5), 75 mM NaCl, 0.1 mM EDTA, 0.1 mM TCEP) immediately prior to use.

### Nucleic Acids

Synthetic oligoribonucleotides were obtained from GE Healthcare Dharmacon (U30, Het30) or TriLink Biotechnologies, Inc. (ppp-Het30), dT30 was obtained from IDT, Inc. ss-dsRNAs were prepared by *in vitro* transcription as previously described^22^. For RNAs containing 4-thiouridine, UTP was replaced with 4-Thio-UTP (TriLink Biotechnologies, Inc.) in the transcription reactions. All RNAs were purified by denaturing PAGE followed by electroelution in an Elutrap device (Schleicher and Schuell).

### Analytical Ultracentrifugation

Protein-RNA interactions were assayed by sedimentation velocity analysis as previously described^51^ in a Beckman Coulter XL-I analytical ultracentrifuge with 260 nm absorbance optics at 20 °C and 50,000 rpm in AU75 buffer. g^(s*) sedimentation coefficient distributions were produced using the time derivative method implemented in DCDT+, version 2.3.2^52^. Binding affinities and stoichiometries were extracted by global fits of multiple data sets to association models using SEDANAL, version 6.01^53^. It was not possible to fit for the sedimentation coefficients of some of the RNA-protein complexes due to cross-correlation with the binding constants. These parameters were fixed at physically reasonably values based on our experience that RNA complexes with PKR have frictional ratios (*f*/*f*_*0*_) near 1.5^14, 22, 36^. Frictional ratios, buffer densities, viscosities, and protein partial specific volumes were calculated using SEDNTERP, version 2011120^54^. RNA partial specific volumes were fixed at 0.55 mL g^−1^.

### UV Crosslinking

UV crosslinking reactions were carried out using RNAs containing 4-thiouridine (see SI Materials and Methods for detailed protocol). Briefly, 5′-^32^P-labeled RNAs were UV crosslinked to PKR constructs containing a TEV cleavage site, treated with TEV protease, and analyzed by SDS-page. The products were visualized on a Typhoon Phosphorimager.

### Activity Assays

PKR autophosphorylation was monitored by incorporation of ^32^P from [γ-^32^P]ATP (Perkin-Elmer). Low molecular weight heparin (4–6 kDa) was obtained from Sigma-Aldrich. Samples contained a fixed concentration of PKR and a variable concentration of activator. The assay was performed in AU75 buffer plus 5 mM MgCl_2_ at 32 °C. Samples were prepared on ice then incubated at 32 °C for 10 minutes prior to initiation of phosphorylation by addition of ATP to a final concentration of 400 µM and 0.25 µCi/µL [γ-^32^P]ATP. Reactions were quenched after 20 min incubation with sample loading buffer and resolved by SDS-PAGE. The gels were dried and exposed to a phosphor screen followed by quantitation on a Typhoon phosphorimager using ImageQuant TL Software (GE Healthcare).

## Electronic supplementary material

Supplementary Information
