# Supplementary material for: Interaction of PKR with single-stranded RNA
Source: Sci Rep. 2017 Jun 13;7:3335. doi: 10.1038/s41598-017-03047-7 (PMC5469796; doi:10.1038/s41598-017-03047-7)
Supplement: Supplementary file 1 — Supplementary Information [file 41598_2017_3047_MOESM1_ESM.pdf]

Interaction of PKR with single-stranded RNA

Christopher B. Mayo[1], and James L. Cole[1,2*]

[1]Department of Molecular and Cell Biology and [2]Department of Chemistry

University of Connecticut, Storrs, Connecticut 06269, USA

Supplementary Information

Supplemental Materials and Methods

Detailed UV Crosslinking Procedure. Crosslinking reactions were carried out using RNAs containing 4-thiouridine. To generate 5′-^{32}P RNA, transcripts were dephosphorylated with calf intestinal alkaline phosphatase (CIP, New England BioLabs) to remove the 5′-triphosphate and phosphorylated with [γ-^{32}P]ATP (Perkin Elmer) and T4 polynucleotide kinase (T4 PNK, New England BioLabs). Spurious autophosphorylation of PKR during the crosslinking reaction due to [γ-^{32}P]ATP contamination from the labeling reaction would complicate interpretation of the results. Therefore, we took extraordinary steps to eliminate free [γ-^{32}P]ATP. The RNA was purified by phenol-chloroform extraction followed by ethanol precipitation. The RNA pellet was resuspended in AU75 buffer containing 10 mM EDTA and buffer exchanged using a series of dilution and concentration steps in a 10,000 MWCO centrifugal filter (Millipore). 10 mM EDTA was included in the final crosslinking reaction to inhibit PKR activity. Crosslinking was performed in AU75 buffer containing 10 mM EDTA. Samples contained 0.5 µM RNA and 1 µM protein in 15 µL. For the dsRBD construct, an additional sample was prepared containing 0.5 µM RNA and 10 µM protein. Samples were incubated on ice 15 minutes prior to UV exposure. An 8 watt ultraviolet lamp (UVP; model 3UV-38) was used to irradiate the samples at 365 nm at a distance of 5 cm for 10 minutes on ice. Samples containing a TEV cleavage site were treated with TEV AcTEV protease (Thermo Fisher Scientific) by combining 5 µL of the crosslinking reaction with 0.5 µL protease and incubating at 32 °C for one hour. Crosslinking reactions were resolved by SDS-PAGE. Gels were fixed in 7.5% acetic acid and stained with SYPRO Orange (ThermoFisher) to visualize the location of protein within the gel. Gels were then dried and exposed to a phosphor screen followed by imaging and quantification on a Typhoon phosphorimager (GE Healthcare).

Supplemental Figure Legends

Figure S1. Thermal denaturation analysis of ssRNAs. (**a**) Predicted secondary structure of Het30. Secondary structure was computed at 20 °C using MFOLD [1]. The free energy of folding is -4.01 kcal/mol. (**b**) Absorbance melting curves. Thermal denaturation was analyzed by monitoring the absorbance change at 260 nm at a heating rate of 0.5 °C/min. The measurements were conducted in 10 mM $NaPO_4$ (pH 7.0) with 0.1 mM EDTA. The heteropolymeric Het30 sequence shows a slight absorbance increase that could be associated with weak secondary structure formation or unstacking but there is no transition which can be assigned to cooperative unfolding. The U30 homopolymer shows a small absorbance decrease which can be attributed to thermal expansion of the solution.

Figure S2. Sedimentation velocity analysis of PKR domain constructs. Measurements were performed in AU75 buffer at 20 °C and 50,000 rpm using interference optics. The data were analyzed using DCDT+ (43) to produce g^(s*) distributions. The distributions are normalized by area. Protein concentrations: PKR, 16 µM; kinase domain constructs, 30 µM; dsRBD, 12 µM.

Figure S3. Sedimentation velocity analysis of 229-kinase binding to Het30 and ppp-Het30. Measurements were performed in AU75 buffer at 20 °C and 50,000 rpm using absorbance detection at 260 nm. The titrations are depicted in normalized overlays of g^(s*) sedimentation coefficient distribution functions. The filled circles correspond to ppp-Het30 and the open circles correspond to Het30. The samples contained 1 µM Het30 or ppp-Het30 (—) and 1 µM Het30 or ppp-Het30 plus 5 eq. (—), 10 eq. (—), and 30 eq. 229-kinase (—).

Figure S4. RNA-independent autophosphorylation of PKR constructs. RNA-independent autophosphorylation of PKR and kinase domain constructs was assayed by measuring the incorporation of ^{32}P over a 20 min time course at 32 °C in AU75 buffer containing 5 mM $MgCl_2$ with 400 µM ATP and 0.25 µCi/µL [γ-^{32}P]ATP. Samples were resolved by SDS-PAGE and stained with Sypro Orange to visualize protein and exposed to a phosphor screen to measure autophosphorylation. (**a**) Top, protein stain; bottom, autophosphorylation. (**b**) Quantitation of ^{32}P incorporation as a function of protein concentration. The data are plotted on a log-log scale. Note that PKR contains ~15 autophosphorylation sites and the full-length enzyme contains more phosphorylation sites per mole than the kinase domain constructs. This difference may contribute to the higher extent of PKR autophosphorylation. In contrast, 242-kinase contains an additional phosphorylation site not present in 229-kinase[2,3] yet incorporates less ^{32}P per mole than 242-kinase. The differences in the extent of phosphorylation can be attributed to a difference in intrinsic activity.

Figure S5. Crosslinking analysis of PKR binding to ss-dsRNA. The protein in the gel corresponding the 4-thiouridine 15-15-15 crosslinking experiment depicted in Fig. 5 were visualized by staining with Sypro Orange. Because crosslinking efficiency is low, the Sypro Orange stain primarily visualizes the free protein whereas the phosphorimager scan in Fig. 5b is specific for ^{32}P-labeled RNA-protein adducts.

Figure S6. Sedimentation velocity analysis of full length binding to ppp-Het30. Measurements were performed in AU75 buffer at 20 °C and 50,000 rpm using absorbance detection at 260 nm. The titrations are depicted in normalized overlays of g^(s*) sedimentation coefficient distribution functions. Sedimentation coefficients calculated using a frictional ratio of

1.5 are shown along the x-axis. The samples contained 1 μM ppp-Het30 (—) and 1 μM ppp-Het30 plus 0.5 eq. (—), 1 eq. (—), 2eq. (—), 5 eq. (—), 10eq (—), and 30 eq. full length PKR (—).

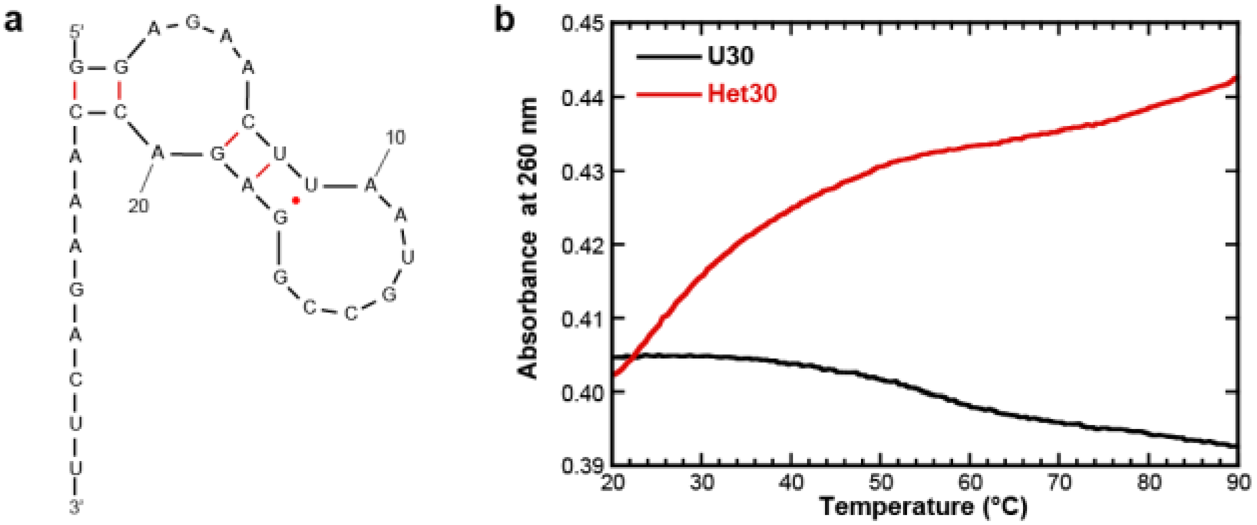
a
5'
3'
10
20
b
U30
Het30
Absorbance at 260 nm
Temperature (°C)
0.45
0.44
0.43
0.42
0.41
0.40
0.39
20
30
40
50
60
70
80
90

Figure S1

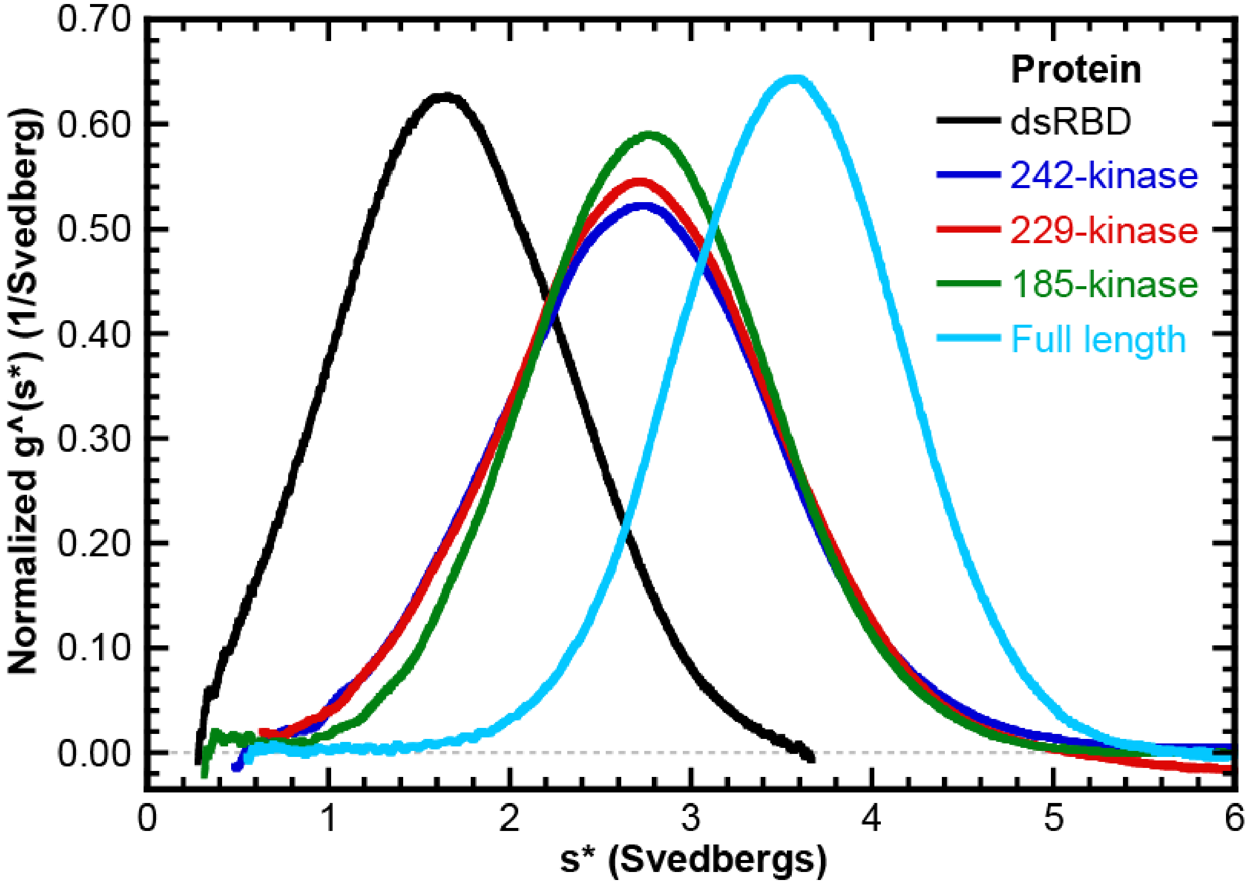

Normalized g^(s*) (1/Svedberg)
s* (Svedbergs)
0.70
0.60
0.50
0.40
0.30
0.20
0.10
0.00
0
1
2
3
4
5
6
Protein
dsRBD
242-kinase
229-kinase
185-kinase
Full length

Figure S2

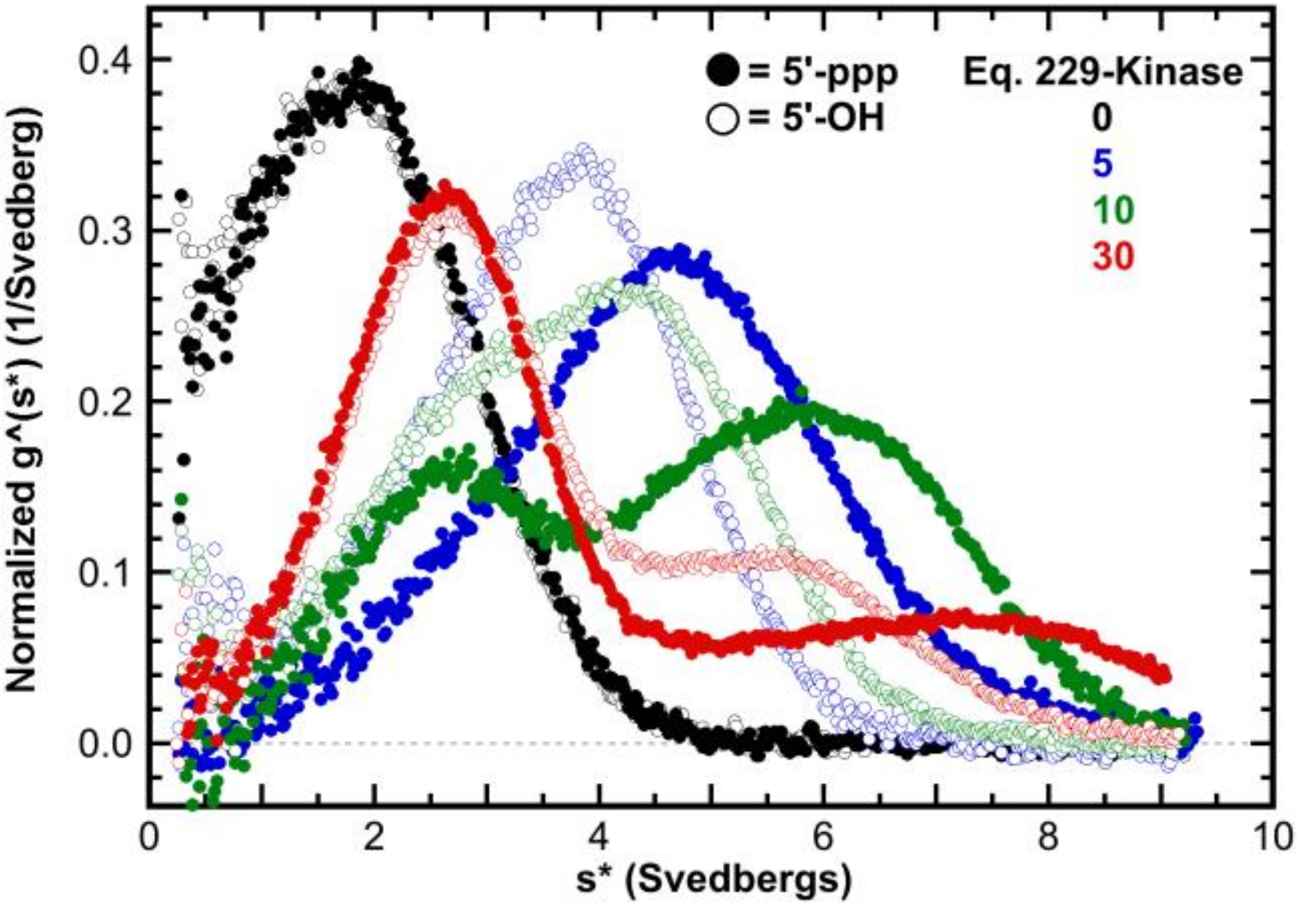

= 5'-ppp
= 5'-OH
Eq. 229-Kinase
0
5
10
30
Normalized g^(s*) (1/Svedberg)
s* (Svedbergs)
0.4
0.3
0.2
0.1
0.0
0
2
4
6
8
10

Figure S3

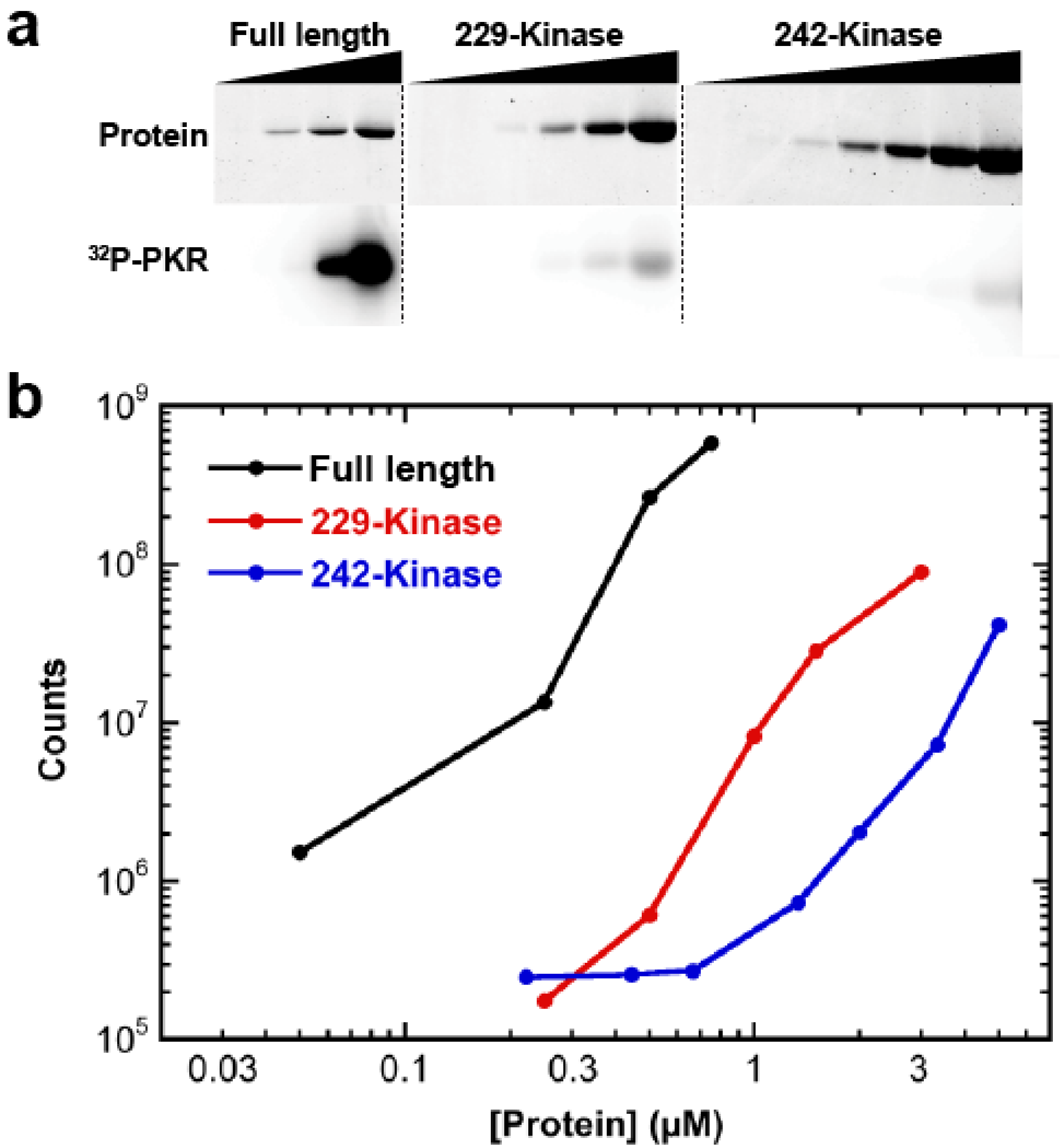
a
Full length
229-Kinase
242-Kinase
Protein
32P-PKR
b
Full length
229-Kinase
242-Kinase
Counts
10^9
10^8
10^7
10^6
10^5
0.03
0.1
0.3
1
3
[Protein] (μM)

Figure S4

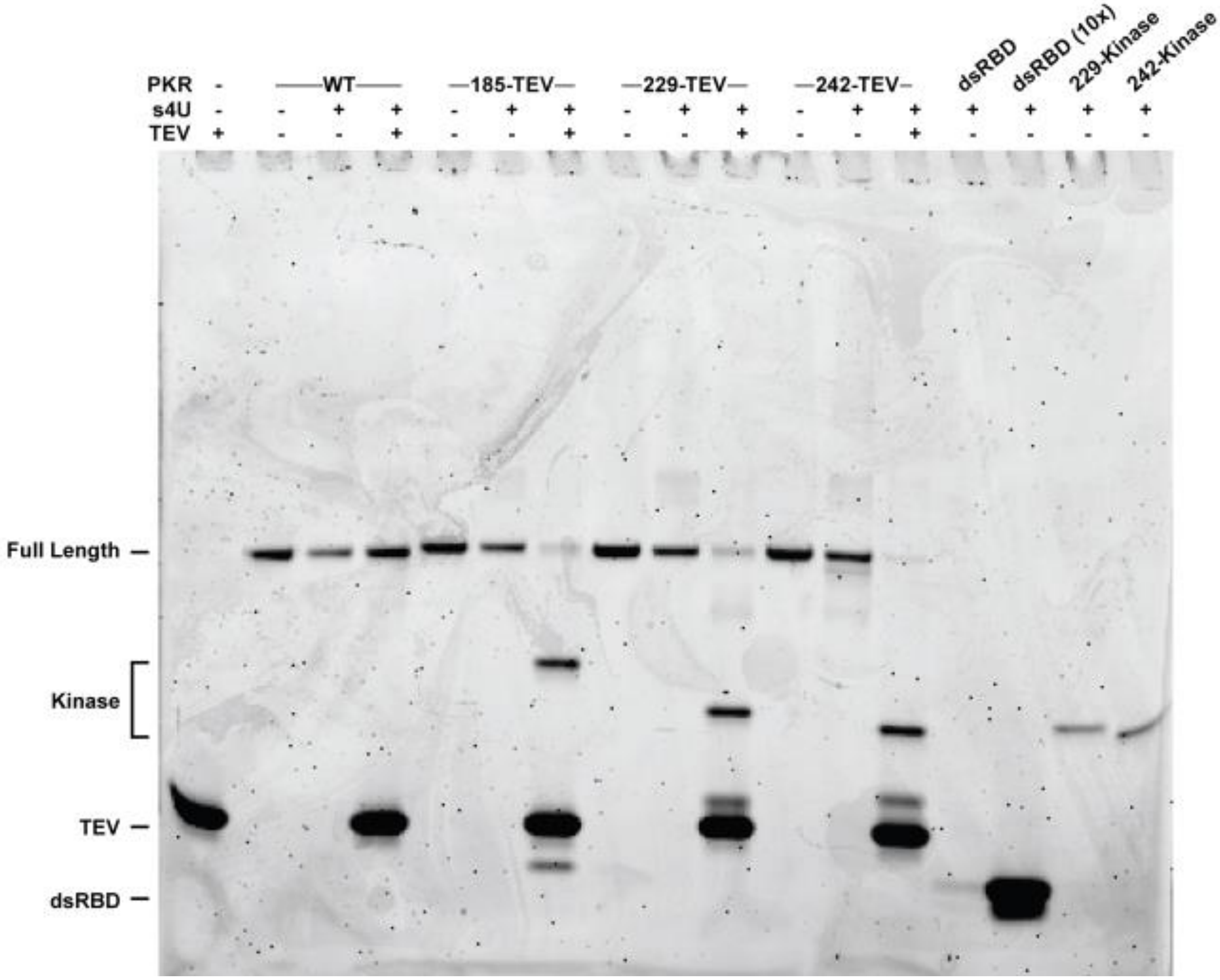
PKR
s4U
TEV
WT
185-TEV
229-TEV
242-TEV
dsRBD
dsRBD (10x)
229-Kinase
242-Kinase
Full Length
Kinase
TEV
dsRBD

Figure S5

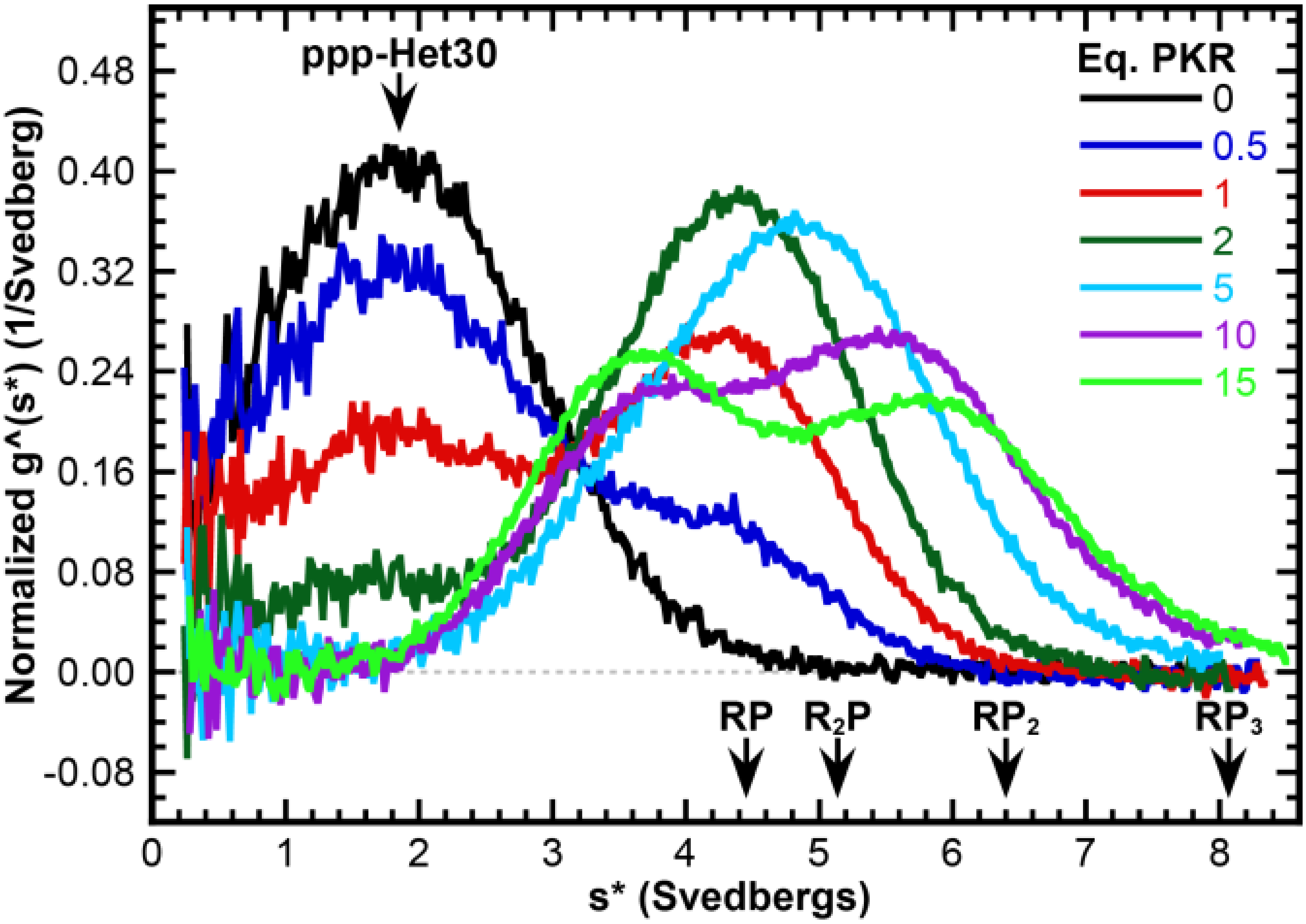

ppp-Het30
Eq. PKR
0
0.5
1
2
5
10
15
RP
R2P
RP2
RP3
Normalized g^(s*) (1/Svedberg)
0.48
0.40
0.32
0.24
0.16
0.08
0.00
-0.08
0
1
2
3
4
5
6
7
8
s* (Svedbergs)

Figure S6

Supplementary Tables

Table S1. Sedimentation velocity analysis of PKR domain constructs.

Protein	Predicted Mass[a]	Fitted Mass	$s_{20,w}$ (Svedbergs)[b]	RMSD[c]
dsRBD	20,262	19,797	1.83	0.0061
242-kinase	35,811	32,830	2.89	0.0207
229-kinase	37,436	38,608	2.91	0.0168
185-kinase	41,907	41,626	2.92	0.0221
Full length	62,095	60,774	3.71	0.0211

Parameters obtained by global nonlinear least square analysis of the sedimentation velocity data to a single ideal species model. Protein concentrations used in the global analysis: dsRBD, 12 µM; kinase domains, 5, 15, 30 µM; Full length PKR, 16 µM

[a] Masses predicted based on amino acid sequence.

[b] Sedimentation coefficient corrected to standard conditions (water at 20 °C).

[c] Root mean square deviation in fringes.

[d] Data from reference [4].

Fits of sedimentation velocity data for ppp-Het30 binding to PKR using alternative models are shown below. The model name refers to the stoichiometric ratio of protein:RNA contained within the saturated complex. The sedimentation coefficients are fixed based on an assumed asymmetric shape of the complex calculated using a frictional ratio (f/f_0) = 1.5. The sedimentation coefficients are reported in Table S2 and the fitted binding equilibria are shown in Tables S3-S5. Based on the RMSD, the best fit model is 2:1.

Table S2. Sedimentation coefficients calculated for a frictional ratio of f/f_0 = 1.5.

s (RP)	s (RP_2)	s (RP_3)	s (R_2P)	s(R_2P_2)
4.40	6.36	8.06	5.15	6.99

Table S3. Sequential binding of multiple PKR to one RNA

Model	Reaction	K_{d1} (μM)	K_{d2} (μM)	K_{d3} (μM)	RMSD
1:1	R + P → RP	273	-	-	0.0359
2:1	R + P → RP + P → RP_2	0.825	3.81	-	0.00876
3:1	R + P → RP + P → RP_2 + P → RP_3	0.856	3.65	252	0.00974

Table S4. Sequential binding of two RNA to one PKR

Model	Reaction	K_{d1} (μM)	K_{d2} (μM)	RMSD
1:2	R + P → RP + R → R_2P	0.372	0	0.0281

Supplementary References

1. Zuker, M. Mfold web server for nucleic acid folding and hybridization prediction. *Nucleic Acids Research* **31,** 3406–3415 (2003).
2. Taylor, D. R. *et al.* Autophosphorylation sites participate in the activation of the double-stranded-RNA-activated protein kinase PKR. *Mol Cell Biol* **16,** 6295–6302 (1996).
3. Wang, D. *et al.* Auto-phosphorylation Represses Protein Kinase R Activity. *Sci Rep* **7,** 44340 (2017).
4. Anderson, E., Pierre-Louis, W. S., Wong, C. J., Lary, J. W. & Cole, J. L. Heparin Activates PKR by Inducing Dimerization. *J Mol Biol* **413,** 973–984 (2011).
